# Effects of a cognitive training on spatial learning and associated functional brain activations

**DOI:** 10.1186/1471-2202-14-73

**Published:** 2013-07-20

**Authors:** Kirsten Hötting, Kathrin Holzschneider, Anna Stenzel, Thomas Wolbers, Brigitte Röder

**Affiliations:** 1 Biological Psychology and Neuropsychology, University of Hamburg, Von-Melle-Park 11, 20146 Hamburg, Germany; 2 German Center for Neurodegenerative Diseases, Leipziger Str. 44, 39120 Magdeburg, Germany

**Keywords:** Exercise, Physical activity, Cognitive training, Cognition, Spatial memory, *f*MRI, Humans, Prevention

## Abstract

**Background:**

Both cognitive and physical exercise have been discussed as promising interventions for healthy cognitive aging. The present study assessed the effects of cognitive training (spatial vs. perceptual training) and physical training (endurance training vs. non-endurance training) on spatial learning and associated brain activation in 33 adults (40–55 years). Spatial learning was assessed with a virtual maze task, and at the same time neural correlates were measured with functional magnetic resonance imaging (*f*MRI).

**Results:**

Only the spatial training improved performance in the maze task. These behavioral gains were accompanied by a decrease in frontal and temporal lobe activity. At posttest, participants of the spatial training group showed lower activity than participants of the perceptual training group in a network of brain regions associated with spatial learning, including the hippocampus and parahippocampal gyrus. No significant differences were observed between the two physical intervention groups.

**Conclusions:**

Functional changes in neural systems associated with spatial navigation can be induced by cognitive interventions and seem to be stronger than effects of physical exercise in middle-aged adults.

## Background

Aging in humans is accompanied by a decline of performance in a wide range of cognitive functions, along with structural and functional changes in several brain regions [1]. Because life expectancy has increased dramatically in industrialized societies within the last decades, it is of particular importance to identify successful strategies for maintaining and enhancing cognitive flexibility and plasticity throughout the lifespan. Mental abilities in childhood [2] and genetic variations [3] are reliable predictors of cognitive functioning in older age. However, the lifestyle of an individual substantially modulates cognitive aging, even in older adults. Different interventions for successful aging have been suggested, for example the control of cardiovascular risk factors [4], caloric restriction [5], or a Mediterranean diet [6], as well as physical exercise [7] and cognitive interventions [8]. The present paper focuses on the effects of a combined physical and cognitive intervention on cognitive functions and their neural correlates.

Prospective epidemiological studies have repeatedly provided evidence for a positive relationship between physical activity and a reduced risk of dementia [9,10]. Cross-sectional studies have demonstrated a positive relationship between physical activity, in particular cardiovascular fitness, and a large number of different cognitive variables [11-15]. In controlled longitudinal intervention studies, the most consistent finding has been a positive effect of cardiovascular fitness on executive functions in older adults [16-19]. These improvements were accompanied by functional changes in associated frontal brain regions, most likely indicating more efficient neuronal processing [16]. Moreover, less age-related decline of grey and white matter volume, especially in the frontal cortex, has been reported after an aerobic exercise training compared to a stretching control training [17].

Recent studies have demonstrated an impact of physical exercise on memory functions as well. Based on their interventional study, Ruscheweyh et al. [20] reported a positive association between the increase in overall physical activity and episodic memory regardless of the exercise intensity. Hötting et al. [21] found a positive correlation between the increase in cardiovascular fitness and verbal memory after a six-month exercise training, suggesting a direct relation between physical exercising success and cognitive gains. Furthermore, older adults with subjective memory impairments have been shown to benefit from physical activity [22]. Moreover, visual-spatial memory [23] and immediate verbal memory [24] in young adults was reported to increase after a few weeks of physical exercise. The latter studies, however, have some methodological weaknesses as they compared data of the physical exercise group to a passive control group [23] or used a pre-posttest design without control group [24]. Thus, these results have to be confirmed in randomized trials.

On the neural level, physical exercise has been shown to increase the hippocampal volume [25] and blood flow in the dentate gyrus [24]. Moreover, changes in the functional connectivity of medial temporal lobe structures have been reported after exercise interventions [26,27].

Cognitive interventions, on the other hand, are specific protocols of tasks developed to improve cognitive functions or to prevent age-related declines. A positive effect of cognitive interventions has been demonstrated in various domains, including working memory (e.g. [28]), processing speed (e.g. [29]), reasoning (e.g. [30]) and executive functions (e.g. [31]). While some authors have postulated that the training effects are rather specific for the practiced domain [8], others have demonstrated considerable transfer to untrained tasks (summarized in [32]).

The effects of an extensive spatial training on spatial skills and associated brain systems have hardly been studies in humans [33-35]. In a study by Lövdén et al. [36] healthy young and healthy old men participated in a four-month virtual reality navigation training. Improved performance in the navigation task compared to a control group was found. Moreover, participants of the spatial training group showed stable hippocampal volumes during and four months after the end of the intervention. By contrast, age-related volume decrements were found for the control group. Thus, a spatial navigation training seems to enhance navigation performance and to protect the hippocampal volume in humans from age-related decline [36]. This conclusion is further supported by a recent longitudinal study in a more natural setting: trainee London taxi drivers who had successfully learned the map of the city of London within four years showed an increase in posterior hippocampal volume compared to participants who failed to acquire this spatial knowledge [37].

Functional brain imaging studies on learning-induced neural plasticity found several different patterns of change after cognitive interventions. An increase in activity after training has been linked to the recruitment of additional cortical tissue or the strengthening of responses within a brain region [38]. Other studies have reported a decrease in brain activity after training. This result has been interpreted as an improved neuronal efficiency and specialization of neural networks [39]. Moreover, shifts of activated brain regions from pre-to posttest have been observed as well. Such functional reallocations have been interpreted as a qualitative change in the cognitive processes recruited for a specific task [39]. To our knowledge, there are no functional MRI studies on the effects of an extensive spatial training on neuronal processing in humans so far. Some spatial navigation studies showed changes with practice within one session. For example, Iaria, Petrides, Dagher, Pike, and Bohobot [34] provided fMRI evidence for a switch in strategy during a spatial navigation learning session. Studies correlating individual differences in performance with fMRI activations during navigation tasks found both positive [40,41] and negative correlations [41] for neural networks associated with spatial navigation. Thus, it seems to depend on the task and instructions used whether improvements in spatial navigation resulted in an increase, a decrease or a reorganization of brain functions. Animal research has suggested that the hippocampus and spatial learning abilities are especially sensitive to both physical exercise and cognitive training. In rodents, wheel running and hippocampus-dependent learning tasks have been shown to enhance neurogenesis [42,43], to increase the release of neurotrophins [44,45] and to improve spatial memory [46,47]. The neuronal mechanisms mediating gains from physical exercise and cognitive stimulation seem to differ: while physical exercise increased especially the proliferation of precursor cells in the subgranular zone of the dentate gyrus, cognitive stimulation promoted the survival of new neurons [48].

Based on these results in rodents, Kempermann [49] proposed the idea that a combination of physical activity and cognitive challenge might be most effective in inducing beneficial and permanent effects on the brain’s structure and function. Results in mice have supported this hypothesis: animals that had access to a running wheel before they were exposed to an enriched environment showed a more pronounced increase in neurogenesis compared to animals exposed to only running or only to an enriched environment [50]. Hence, it could be hypothesized that physical activity might boost the effects of a cognitive intervention in humans, too. To our knowledge only one study has tried to test this hypothesis in humans: Fabre et al. [51] showed that a combination of an aerobic endurance training and a cognitive training targeting various cognitive functions (e.g. memory, attention, spatial skills) was more effective in improving cognitive variables in older adults than any of the trainings alone or no training at all. Middle-aged adults have hardly been included in such studies. Thus, it is unknown whether cognitive, physical or a combined cognitive and physical intervention is beneficial in younger age groups as well. Interventions targeting middle-aged individuals, however, might be especially important to prevent age-related cognitive decline, since midlife physical activity has been found to determine brain structure and function at later ages [52].

In the present controlled interventional study, sedentary 40 to 55 year old adults were randomly assigned to either a six-month long aerobic endurance training (‘cycling’) or a non-endurance control training (‘stretching’). In addition, participants took part in a cognitive intervention during the last month of the physical intervention. They were randomly assigned to either a spatial training (‘spatial training’) or a non-spatial control training (‘perceptual training’) group. Spatial learning and functional brain activation (*f*MRI) were measured during a spatial maze task (see Wolbers et al. [53]) before and after the interventions. Recently, we reported that cardiovascular fitness modulated brain activation during successful spatial learning [54]. In the present report we focus on the effects of the physical and cognitive interventions on spatial learning and associated brain activity. Based on previous results in animals [50] and humans [51] it was hypothesized that both, a physical training and a cognitive training, has beneficial effects on spatial learning. We predicted additive effects, both at the behavioral level and in neural structures associated with spatial learning, including the hippocampus and parahippocampal gyrus (e.g. [55]), the inferior and superior parietal cortex and the retrosplenial cortex (e.g. [56,57]), the caudate nucleus (e.g. [58]), and the cuneus and medial frontal gyrus (e.g. [59]). Due to inconsistent reports in the literature, we did not make explicit predictions about the direction of training induced changes in the BOLD signal.

## Methods

### Participants

Participants were healthy adults between 40 and 55 years of age. Only individuals with a rather sedentary lifestyle during the past five years (i.e. less than two physical exercise sessions per month) were invited to take part. This study was part of a larger controlled interventional study that comprised a total of 106 participants [21]. Forty-seven of them took part in the *f*MRI-experiment reported here. An initial extensive medical examination confirmed that participants were free of severe medical conditions. One participant was excluded after the medical check. No neurological or psychiatric disorders were reported. Participants verbal IQ was assessed with a multiple choice vocabulary test (MWT; [60]) to control for any possible IQ differences between the experimental and the control group. During this test, participants had to identify valid German words intermixed with pronounceable pseudo-words. Item difficulty was manipulated by using valid German words with decreasing familiarity. The MWT-IQ has been shown to correlate well with the general IQ in healthy adults [61]. Eleven participants dropped out during the exercising phase (*n* = 4 cycling; *n* = 7 stretching). Two *f*MRI-datasets were incomplete due to technical problems during data acquisition. Hence, data analysis in this report is based on *n* = 33 participants. These participants had a mean age of 48.9 years (SD = 4.0) and 17 were female. The four subgroups, based on the physical and cognitive trainings (cycling/ spatial: *n* = 8, cycling/ perceptual: *n* = 8, stretching/ spatial: *n* = 9, stretching/ perceptual: *n* = 8), and the group of dropouts (*n* = 11) did not differ with respect to age (*F*(4, 39) = 2.12; *p* = .096), sex (*χ*^*2*^ (4) = 1.34, *p* = 0.85), verbal IQ (*F*(4, 39) = 0.89; *p* = .477) and years of education (*F*(4, 39) = 0.73; *p* = .577). Demographic data for the four resulting subgroups and the group of dropouts are presented in Table 1. All participants were right-handed and had normal or corrected-to-normal vision. They signed a written informed consent and received a monetary compensation for participation. The study was approved by the ethics committee of the German Psychological Society (Deutsche Gesellschaft für Psychologie; DGPs).

**Table 1:** Demographic data for the experimental subgroups

	**Cycling/spatial**	**Cycling/perceptual**	**Stretching/spatial**	**Stretching/perceptual**	**Dropouts**
*n*	8	8	9	8	11
Age *M* (*SD*)	50.25 (4.20)	49.00 (4.28)	50.22 (2.91)	46.00 (3.89)	46.55 (4.85)
female/ male	4/4	5/3	4/5	4/4	4/7
verbal IQ^a)^*M* (*SD*)	126.50 (12.35)	121.50 (10.07)	125.00 (13.72)	116.13 (15.26)	121.18 (9.24)
years of education *M (SD)*	14.00 (4.28)	15.25(3.96)	16.56 (2.96)	16.38 (3.11)	14.82 (3.84)

*M* Mean, *SD* standard deviation*.*

^a)^ assessed with a German multiple choice vocabulary test (Mehrfachwahl-Wortschatz-Intelligenztest, MWT; [60]).

### Assessments

#### Assessment of cardiovascular fitness

Individual cardiovascular fitness was assessed during a three-minute incremental exercise test on a cycle ergometer (Lode Excalibur Sport 1000 W, Lode Medical Technology, Groningen, Netherlands) before (pretest/ T0) and after (posttest/ T1) the interventions. Maximal oxygen uptake (VO_2_peak) during the incremental exercise test was taken as a measurement of individual cardiovascular fitness. Furthermore, the individual aerobic-anaerobic threshold was determined for each participant. Heart rate at 85% of the workload at this threshold at pretest was appointed the ideal aerobic heart rate for the exercise training in the cycling group.

#### Assessment of spatial learning in a virtual maze task

A virtual maze task was used for the assessment of spatial learning capacities at pre- and at posttest. The stimuli and procedure of this task have been described in detail elsewhere [53,54]. In short: Using the software “Blitz 3D” (Blitz Research, Auckland, New Zealand), a virtual 3D-reality was constructed, consisting of three intersections with buildings serving as landmarks. In the encoding phases, participants were passively moved through the environment from a first-person perspective. They repeatedly traveled along each path and had to encode nine different landmarks. Participants were instructed to mentally construct an allocentric, aerial view of the environment. To successfully build a viewpoint-independent representation of the environment, participants had to integrate visual motion cues with spatial information. During the experimental retrieval phase 12 pairs of the buildings, which had been encountered during the encoding phase, were shown in a randomized order. Stimuli were presented using the software “Presentation” (Version 11.0, Neurobehavioral Systems, Albany, CA, USA). Participants` task was to imagine standing within the environment, facing the top building and to indicate the relative position of the bottom building. Responses were given by pressing a button on an MR-compatible button box with index (left), middle (behind) and ring finger (right). Six experimental sessions, comprising one encoding phase followed by one retrieval phase each, were run.

Additionally, non-spatial control conditions for the experimental encoding and retrieval phases were run. They were carefully matched for visual input but did not require spatial learning or recall: Participants were moved along a single virtual corridor with varying buildings presented at both ends. A total of nine different buildings, not used in the experimental encoding phase, were shown. Sets of buildings assigned to the experimental vs. control conditions, however, were counterbalanced across participants. Thus, the visual stimulation did not differ between the experimental and control conditions. During the control condition for the encoding phase, a small black cube was placed in front of some of the buildings. Participants were instructed to silently list the number of encountered cubes to capture working memory capacities and avoid silent rehearsing of the preceding experimental environment. During the control condition for the retrieval phase, participants saw 12 pairs of buildings and were asked to indicate by pressing a button whether the two buildings were identical or different. The three control sessions, comprising one control encoding and one control retrieval phase each, were placed at the beginning, in the middle and at the end of the experiment.

The virtual maze task was performed inside the scanner prior and after the physical and cognitive interventions. Scanning was performed throughout each session and *f*MRI data were collapsed across the six experimental encoding and three control encoding sessions, respectively, separately for pre- and posttest (see also Data analysis).

#### Assessment of verbal learning and executive functions

To test whether effects of the spatial training induce specific changes in spatial cognition or show a transfer to other domains, two paper-and-pencil-tests assessing verbal learning and executive functions, respectively, were applied at pre- and posttest. Verbal learning was assessed with the German equivalent of the Auditory Verbal Learning Test [62], and executive functions were assessed with the Stroop task [63,64].

#### *f*MRI data acquisition

MR scanning was performed on a 3T-MRI scanner (TRIO; Siemens AG, Munich, Germany) using a standard head coil. A T2*-sensitive echo planar imaging sequence (TR 2420 ms; TE 30 ms; FOV, 216 mm × 216 mm) was used to acquire 37 axial slices (voxel size 3 × 3 × 3 mm). Moreover, a T1-sensitive standard MPRAGE sequence was used to acquire a whole head structural image (1 mm × 1mm × 1mm).

Stimuli were projected with a video projector onto a screen positioned at the top of the head coil. This screen was reflected by a small mirror attached to the head coil (9.5 × 11.5 cm, 45° angle), right above participants eyes. Participants lay on their backs and watched the experiment in the mirror. All had normal vision or wore MR-compatible correction glasses during scanning; none had difficulties seeing the experimental stimuli. Participants’ heads were stabilized with foam pads to minimize head movements.

### Interventions

This study was a longitudinal controlled interventional study. Participants were randomly assigned to either an aerobic endurance training (Indoor Cycling, ‘cycling group’) or a non-endurance training (Stretching and Coordination, ‘stretching group’). Both groups exercised twice a week for six months. To investigate potential interactions of a physical and a cognitive intervention, one half of the participants of each exercise group received a spatial training; the other half participated in a perceptual training. Cognitive trainings took place in six individual, computerized training sessions during the last month of the physical intervention. The duration of each cognitive training session was approximately 45 minutes. The interventions are described in detail below.

#### Physical trainings

##### Cycling training

The aerobic endurance training was an indoor-cycling training on stationary bicycles. After a warm-up phase, training intensity was based on individual results of the incremental exercise test (see Assessment of cardiovascular fitness). Participants were required to keep their heart rate within this range for approximately 45 minutes. Subsequently, a cool-down phase followed. Cycling training is known to improve cardiovascular fitness which was confirmed in the present study: there was a significant increase in VO_2_peak from pre-to posttest in the cycling group (*t*(15) = 3.59; *p* = .003; see Table 2).

**Table 2:** **Cardiovascular fitness (VO**_**2**_**peak in ml/kg/min)) at the pre- and posttest for the two groups of the physical intervention**

	**Cycling**	**Stretching**
*n*	16	17
pretest/ T0 *M*(*SD*)	29.97 (4.81)	30.72 (3.84)
posttest/ T1 *M*(*SD*)	34.83 (5.75)	32.09 (3.95)

*M* mean, *SD* standard deviation.

##### Stretching training

The stretching and coordination training was intended to not affect cardiovascular fitness while holding other variables like social interactions, instructors, schedule, etc. as similar as possible to the cycling training. Each session started with a short warm-up phase, followed by stretching-, strengthening-, coordination- and relaxation-exercises. The intensity of these exercises was far below 85% of the aerobic-anaerobic threshold and there was no significant change in VO_2_peak from pre-to posttest (*t*(13) = 1.08; *p* = .30; see Table 2).

#### Cognitive trainings

##### Spatial training

The spatial training was intended to improve aspects of spatial cognition that were required in the spatial maze task used to determine spatial learning capacities in the present study. Therefore, two different tasks were introduced: a viewpoint shift task and a path integration task.

**Figure 1:**
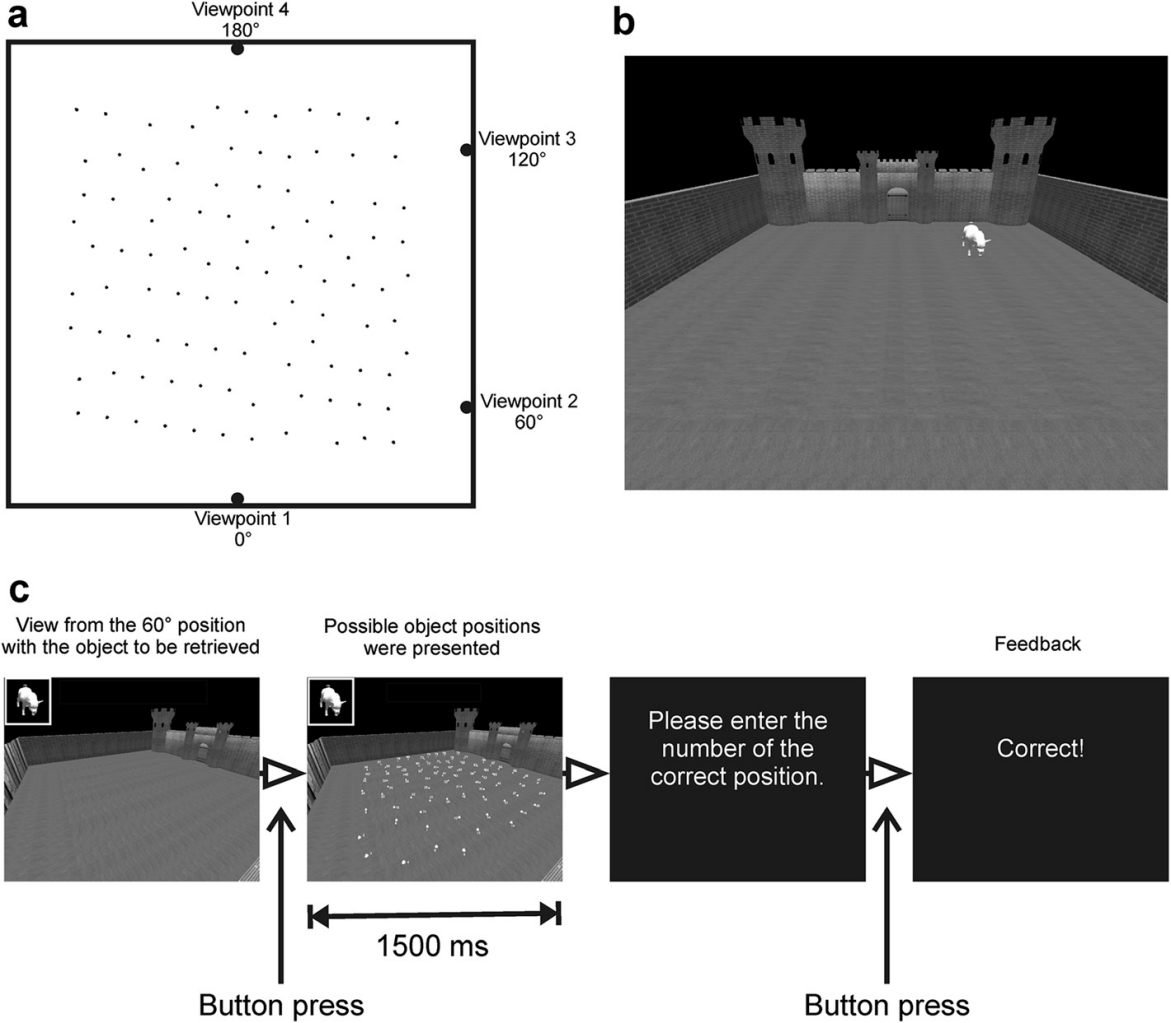
**Viewpoint shift training. a)** Schematic aerial view of the courtyard with possible object positions. During learning, participants looked into the courtyard either from viewpoint 1 or 2. An example for viewpoint 1 is shown in **b)**. During retrieval, four different viewpoints were used. **c)** Stimuli and trial timing during retrieval. The short presentation time for the possible object positions was chosen to prevent any strategic counting of positions. Moreover, different arrangements of possible positions and numberings were used across trials; thus, remembering the correct number of an object within a trial did not allow a correct localization of the same object in the next trial. Participants got feedback after each response indicating whether their response was correct, close to the mark or wrong.

In the viewpoint shift task different objects were sequentially presented in a virtual room on a computer screen (see Figure 1) and participants were asked to encode the objects’ positions. After a short distraction phase, the objects’ positions had to be retrieved from the same or a different perspective. Retrieving objects from a different perspective (i.e. a shifted viewpoint) has previously been shown to require allocentric memory skills and to depend on the hippocampus [65].

The virtual room consisted of a courtyard surrounded by walls with distinct textures or features. Thus, these walls could be used as landmarks to memorize the objects’ positions. Objects were either animals, vehicles, furniture, plants, technical devices or home appliances. A new category was introduced on each day of training to avoid proactive interference. The assignment of categories to days was randomized across participants. During learning, participants looked into the courtyard from one of two possible viewpoints, either from 0° or 60° relative to the centroid of the area (see Figure 1a and b). The viewpoint was held constant across all learning trials within a day and alternated between days. Five objects were sequentially shown for 3 sec; each with an interstimulus interval of 1 sec. Participants had to solve simple arithmetic problems for 20 sec between encoding and retrieval to prevent rehearsal strategies. During the following retrieval phase, participants saw the courtyard either from the same viewpoint as during encoding or from one of three other possible shifted viewpoints (0°, 60°, 120° or 180°, see Figure 1a &1c). An object was shown and participants were asked to retrieve its position, fixate that position and press a button as fast as possible to assess the retrieval time. The touch of the button triggered the presentation of 79 possible object positions within the courtyard each labeled with a number. Participants were prompted to enter the digit of the retrieved position on a standard computer keyboard. After retrieving all five objects, a new learning phase started. This learning-retrieval cycle was repeated for 20 minutes or until all five positions were retrieved correctly. The percentage of correct responses for each training day is reported in the following.

Path integration is the ability to keep track of changes in orientation and position during movement through monitoring motion cues [66]. The following task was assumed to improve participants’ path integration abilities in a virtual environment by using optic flow information.

**Figure 2:**
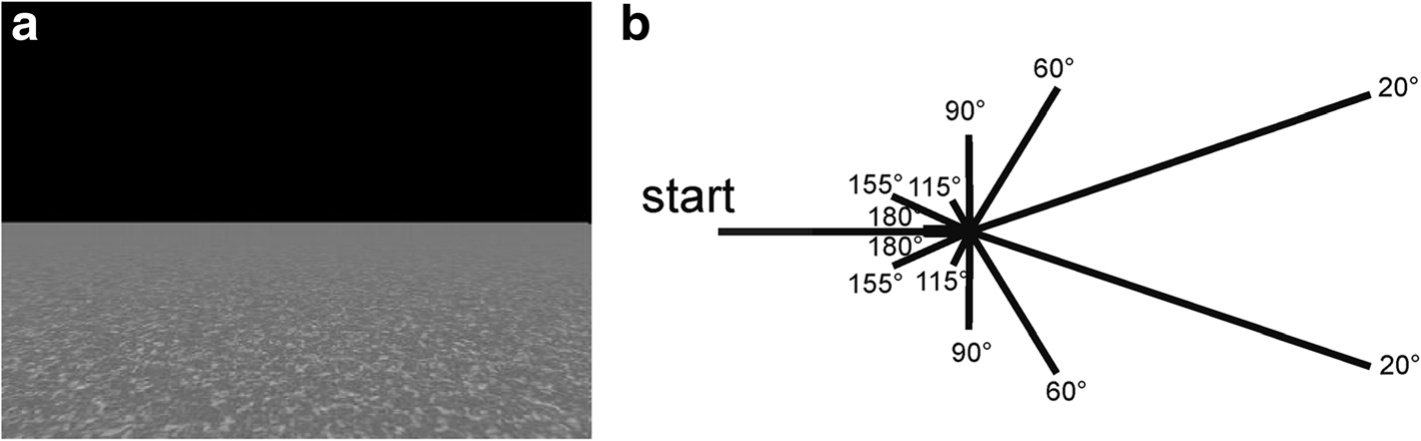
**Path integration training. a)** Virtual plane from the ego-perspective. **b)** Schematic drawing of the possible paths. Feedback was given after each trial (‘correct’ for pointing errors less than 10°, ‘near the mark’ for pointing errors less than 20° and ‘wrong’ for a deviation between correct response and given response of more than 20°).

Participants saw a virtual plane (uniform surface without landmarks) from a first-person perspective (see Figure 2a). Optic flow information was provided as dynamically changing floor texture. In the ego perspective, participants were passively moved straight forward (translation 1), made a turn (rotation) and moved further (translation 2). Each trial started with the presentation of the virtual environment for 1 sec and a translation with a duration of 5 sec. After a delay of 0.5 sec, a rotation (20°/sec) of 20°, 60°, 90°, 115°, 155° or 180° was carried out, either to the left or to the right. The second translation was initiated 0.5 sec after the end of the rotation and varied in length (13.5 to 16.5 sec; see Figure 2b for all possible paths). After another period of 0.5 sec participants were prompted to point with a joystick (Attack™ 3, Logitech) towards the starting point. The twelve possible paths were repeatedly presented in a randomized order until a training duration of 10 minutes was reached. The pointing error was recorded for each trial; the mean pointing error for each training day is reported in the result section.

Although the spatial training targeted cognitive functions that were important for solving the maze task, it is important to note that the stimuli and tasks used during the spatial training clearly differed from those of the maze task.

##### Perceptual training

The perceptual training was introduced as a control condition to avoid unspecific effects of a cognitive intervention (e.g. time enrolled in the study, interacting with research staff and practicing a computerized task). Participants in the perceptual training group practiced visual discrimination of Vernier-stimuli. They saw two lines on an oscilloscope screen, side by side or one above the other, and indicated by pressing a button whether the left line was displaced above or below the right line (horizontal condition) or whether the upper line was displaced left or right relative to the lower line (vertical condition). This training was repeated for 30 minutes. The training of visual discrimination abilities is known to induce plasticity in visual brain areas [67] and to be independent of the medial temporal lobe [68]. Based on the literature it was assumed that perceptual training would not affect spatial skills. Performance in the perceptual training task improved across training sessions; the rate of improvement did not differ between the two exercise groups (cycling vs. stretching).

### Data analysis

#### Behavioral data

Behavioral data was analyzed using R (Version 2.14.0, [69]). First, we tested whether improvements across training sessions within the spatial training group were modulated by the physical intervention. Therefore, results of the viewpoint shift training and the path integration training for each training day were entered into an ANOVA with the factors Cognitive Training Day (1–6) and Physical Training (cycling/ stretching). The main purpose of the present study was to investigate whether effects of the spatial training generalized to the virtual maze task used in the *f*MRI sessions and whether these possible transfer effects of the cognitive training were modulated by the type of physical training. Therefore, the percentage of correct responses in the retrieval phase of the virtual maze task was calculated per session and per participant and entered into a repeated measurements ANOVA with the factors Time (T0/ T1), Session (1–6), Physical Training (cycling/stretching) and Cognitive Training (spatial/ perceptual). The significance threshold was set to *p* < .05 for all analyses. *P*-values were Huynh-Feldt-corrected when the sphericity assumptions were violated for repeated-measures factors with more than two levels.

#### *f*MRI data

##### Preprocessing

Image preprocessing and statistical analyses were performed using SPM5 (http://www.fil.ion.ucl.ac.uk/spm/software/spm5). The first four volumes per session were discarded. The remaining volumes were realigned to the first volume that was included. The T1-weighted anatomical image was coregistered to the first included functional volume and segmented using the unified segmentation approach [70] as implemented in SPM5. Thereafter, all functional images were spatially normalized to MNI-space using the normalization parameters obtained from the segmentation procedure and smoothed with an 8 mm full-width at half-maximum Gaussian kernel.

##### Statistical analysis

On the first level, a participant-specific design matrix was created for each time point separately. For each participant and time point contrast images, contrasting experimental and control encoding phases, were calculated and entered into group analyses.

On the second-level, one-sample *t*-Tests were performed, separately for the pre- and the posttest, to test for differences in brain activations between the spatial learning condition and the control condition for the entire group. To determine changes in brain activation over time a Flexible-Factorial-Model was established, including the factors Time (T0/ T1), Physical Training (cycling/stretching), and Cognitive Training (spatial/ perceptual). The main effects of each of these factors, as well as the interactions of Time × Physical Training, Time × Cognitive Training, and Physical Training × Cognitive Training, were calculated with this model. In addition, a separate Full-Factorial-Model was calculated for the pre- and posttest, in order to test for an interaction of Physical training and Cognitive training at T0 and T1, respectively. Changes in activations from pre- to posttest were investigated with paired *t*-Tests. Two-sample-*t*-Tests were run to test for group differences. Except for the one-sample *t* -Tests, which were performed with a Family Wise Error (FWE)-corrected threshold, all other analyses were performed with a False Discovery Rate (FDR)-corrected threshold (*p* < .05), corrected for the whole volume.

Additionally, based on previous studies using a similar spatial learning paradigm [53,71], regions of interest (ROI) analyses were conducted for the medial frontal gyrus, the inferior parietal cortex, the superior parietal cortex, the cuneus, the retrosplenial cortex, the parahippocampal gyrus, the hippocampus and the caudate nucleus. Except for the retrosplenial cortex all regions were determined using the WFU pickatlas, Version 2.4 [72], which was used to perform the small volume corrected analyses as well. The Talairach daemon (TD) atlas [72] was used to determine the medial frontal gyrus, all other regions were determined using the automatic anatomic labeling (AAL) atlas [73]. The retrosplenial cortex was defined as a spherical search volume with a 15 mm radius, positioned at the MNI coordinates +/−15, -45, 9 (Talairach coordinates +/−8, -44, 11; see Brede database; [74]). For each ROI, a small volume corrected analysis, as implemented in SPM, was run. This involves a restriction of the voxel-wise comparison to the defined region and, thus, controls for multiple comparisons within that region. An FDR-corrected threshold (*p* < .05), adjusted for the respective region, was applied. For all analyses, only clusters comprising at least ten significant voxels (after correction for multiple comparisons) are reported.

## Results

### Spatial training: Behavioral data across training sessions

**Figure 3:**
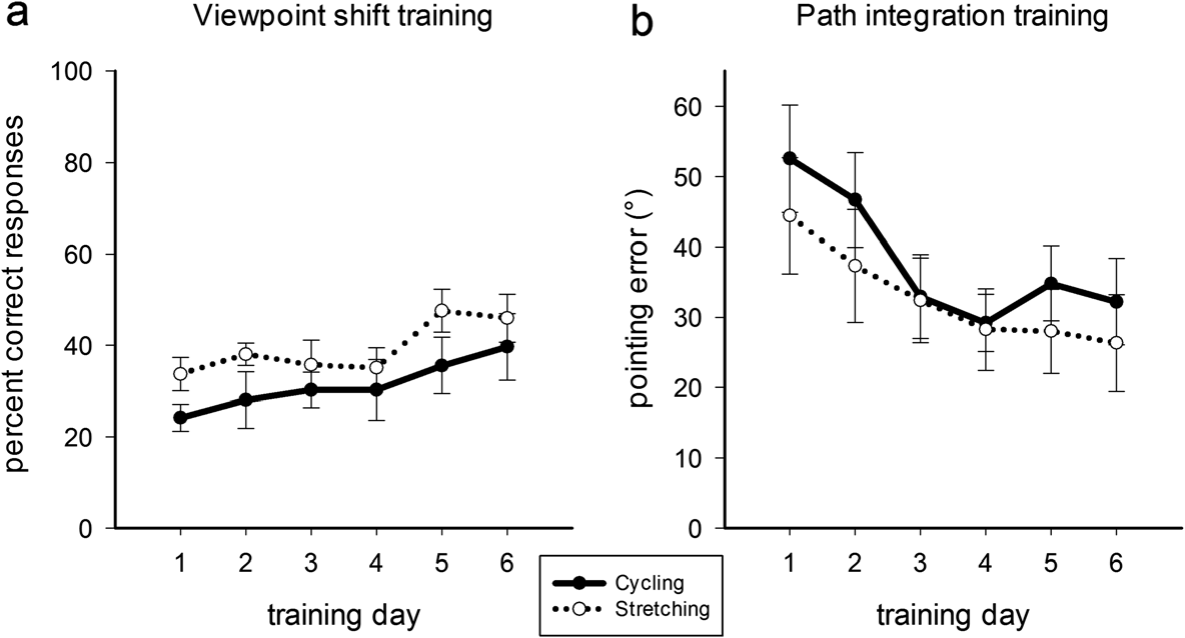
**Learning curves for the viewpoint shift training (a) and the path integration training (b) across training days separately for the cycling group (solid line) and the stretching group (dashed line).** Chance level in the viewpoint shift task was 1.3%. Error bars depict +/− 1 standard error.

As seen in Figure 3a, participants improved in the viewpoint shift task across training sessions. The number of correct responses increased from the first to the sixth training day (main effect Cognitive Training Day *F*(5, 75) = 5.52, *p* < 0.001, *η*^*2*^_*G*_ = 0.118). The learning rate did not differ between the cycling group and the stretching group (Cognitive Training Day × Physical Training *F*(5, 75) = 0.38, *p* = 0.86, *η*^*2*^_*G*_ = 0.009).

A similar pattern of results was seen for the path integration training. As depicted in Figure 3b, participants committed less pointing errors with increasing number of training days. Again, there was no difference between the cycling and the stretching group (main effect Cognitive Training Day *F*(5, 75) = 6.39, *p* = 0.001, *η*^*2*^_*G*_ = 0.139; Cognitive Training Day × Physical Training *F* (5, 75) = 0.35, *p* = 0.79, *η*^*2*^_*G*_ = 0.009).^a^

### Virtual maze task: Behavioral data

**Figure 4:**
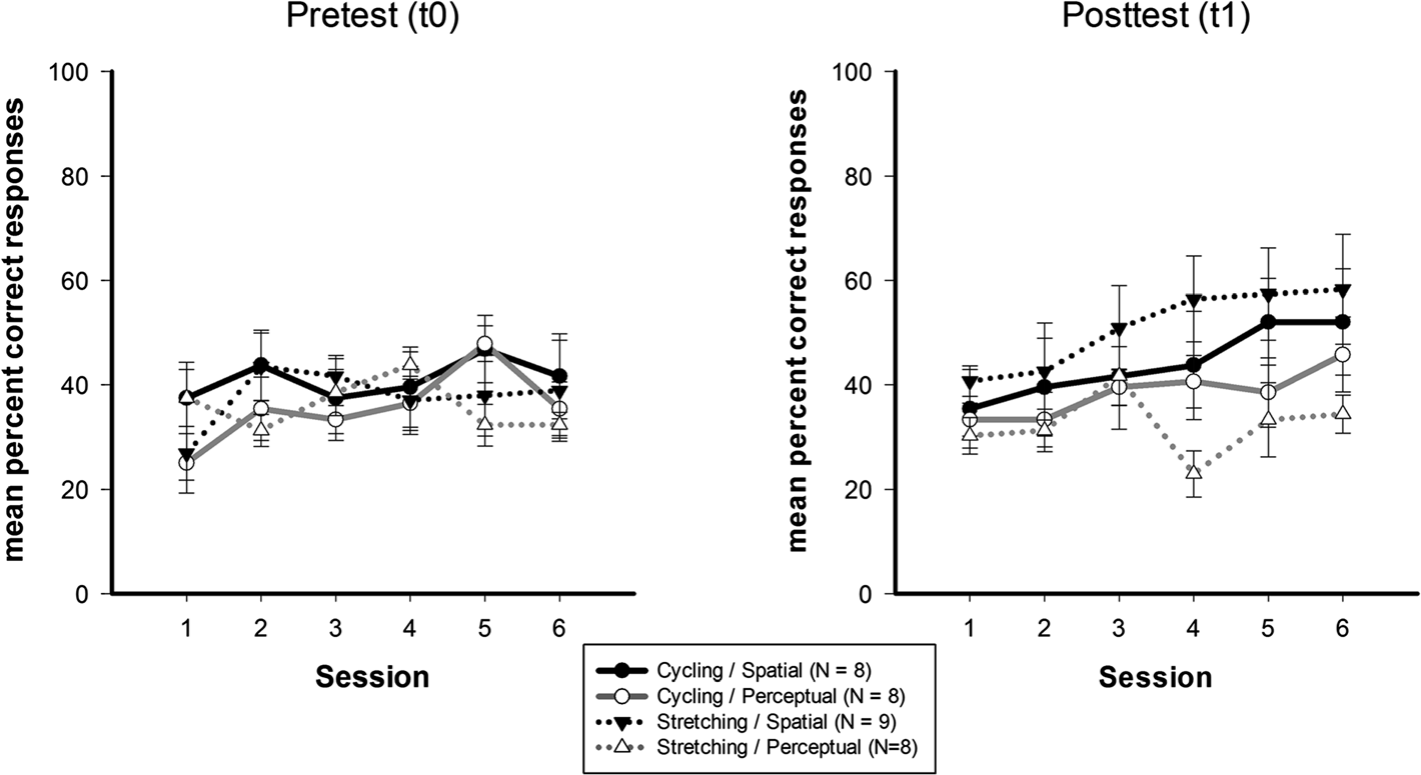
**Mean percent correct responses for the virtual maze task administered during *f*MRI scanning.** Pretest (left) and the posttest (right) data are shown for the four subgroups cycling/ spatial (black/ solid line), cycling/ perceptual (gray/ solid line), stretching/ spatial (black/ dashed line), stretching/ perceptual (gray/ dashed line). Chance level in the maze task was 33.3%. Error bars depict +/− 1 standard error.

From pre- to posttest, there was a trend for the spatial training group to improve performance in the virtual maze task more than the perceptual training group (see Figure 4; Time x Cognitive Training: *F*(1, 29) = 3.18; *p* = .085; *η*^*2*^_*G*_ = .015; Time x Cognitive Training × Physical Training: *F*(1, 29) = 2.92; *p* = .098; *η*^*2*^_*G*_ = .014) and, all in all, the spatial training group showed an overall superior performance (main effect of Cognitive Training: *F*(1, 29) = 3.14; *p* = .087; *η*^*2*^_*G*_ = .047). Separate ANOVAs for the pre- and posttest revealed that participants showed reliable spatial learning of the virtual environment only at posttest, indicated by a gradual increase of correct responses from session 1 to 6 (main effect of Session: *F*(5, 145) = 5.11; *p* < .001; *η*^*2*^_*G*_ = .051). Separate analyses for the cognitive training groups at posttest showed reliable spatial learning for the spatial training group (main effect of Session: *F*(5, 75) = 6.16; *p* < .001; *η*^*2*^_*G*_ = .067), whereas in the perceptual training group the percentage of correct responses did not significantly increase throughout the experiment (main effect of Session: *F*(5, 70) = 1.51; *p* = .218; *η*^*2*^_*G*_ = .074). There was a trend towards better performance of the spatial training group compared to the perceptual training group at posttest (main effect of Cognitive Training at T1: *F*(1, 29) = 4.13; *p* = .051; *η*^*2*^_*G*_ = .090). The type of physical intervention, however, did not significantly influence spatial learning performance (main effect of Physical Training: *F*(1, 29) = .02; *p* = .897; *η*^*2*^_*G*_ < .001; main effect of Physical Training at T1: *F*(1, 29) = .005; *p* = .946; *η*^*2*^_*G*_ < .001; Cognitive Training × Physical Training at T1: *F*(1, 29) = 1.190; *p* = .284; *η*^*2*^_*G*_ = .028).

### Verbal learning and executive functions

Effects of the spatial training did not generalize to verbal learning capacities and executive functions. Although all participants improved from pre- to posttest, both in the Auditory Verbal Learning Task and the Stroop task (main effect of Time all *F* > 7, *p* < 0.01), improvements were not modulated by the kind of cognitive training^b^ (Time × Cognitive Training all *F* < 2, *p* > 0.17).

### Virtual maze task: *f*MRI data

**Figure 5:**
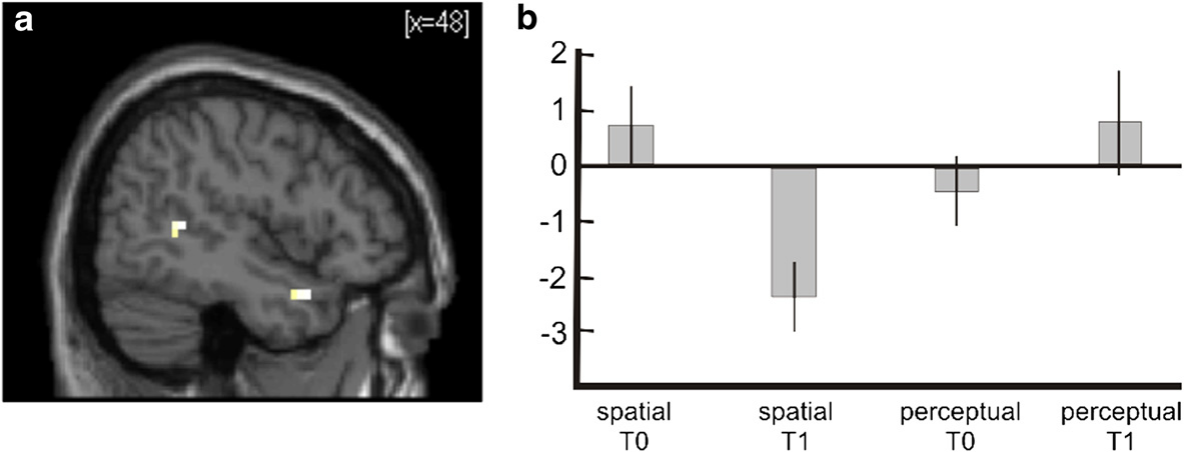
**Interaction between Time and Cognitive Training. a)** Statistical parametric map showing right superior temporal lobe activation for the contrast spatial/ T0 > spatial/ T1 and perceptual/ T0 < perceptual/T1. Activation is superimposed on a normalized single-subject T1-template available in SPM5. **b)** Contrast estimates for the contrast ‘encoding experimental > encoding control’ in a superior temporal peak voxel (x = 48, y = 12, z = −25), indication decreasing activation in the spatial training group from pre- (T0) to posttest (T1). Y-scale in arbitrary units. Error bars depict 90% CI.

**Figure 6:**
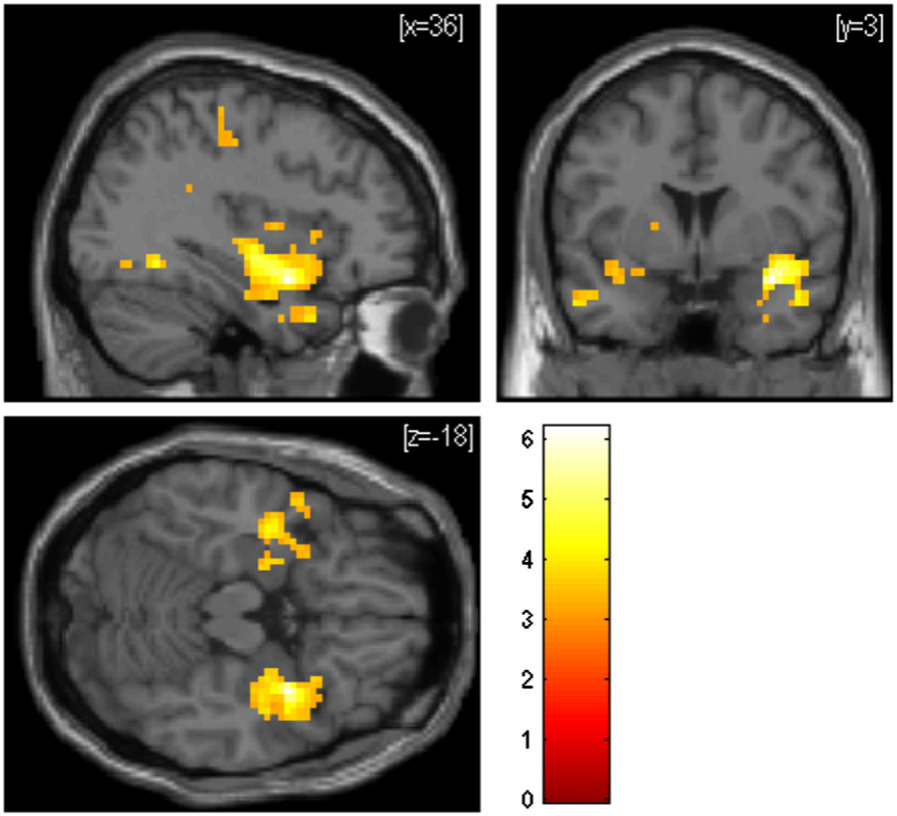
**Statistical parametric maps showing brain activations that were significantly lower in the spatial training group than in the perceptual training group (FDR-corrected; p < .05) during spatial learning (contrast ‘encoding experimental > encoding control’) at posttest.** Activation is superimposed on a normalized single-subject T1-template available in SPM5. Color scale indicates T-scores.

Brain activation associated with spatial learning was assessed separately for pre- and posttest sessions by contrasting activity during experimental encoding sessions with activity during control encoding sessions. These contrasts showed activations in a broad network of brain areas (see Tables 3 and 4). At both time points the activations encompassed the areas a priori defined as regions of interest, including the medial frontal gyrus, the inferior and superior parietal cortex, the retrosplenial cortex, and caudate nucleus, as well as additional regions in the frontal, parietal and temporal cortex. We asked whether these activations changed differentially from pre- to posttest, depending on both the physical and the cognitive intervention. These analyses showed that activations during spatial learning developed differently from pre- to posttest in the cognitive training groups (Time × Cognitive Training interaction; see Table 5). In the spatial learning group, activations decreased in the right middle temporal gyrus, superior temporal gyrus and in an additional cluster in the right medial temporal lobe. By contrast, similar changes in these regions were not observed for the perceptual training group (see Figure 5). A direct comparison between the spatial and the perceptual training group at posttest revealed significantly lower activity in a network of frontal, parietal and temporal regions, and in the hippocampus and parahippocampal gyrus in the spatial training group compared to the perceptual training group (see Table 6 and Figure 6). By contrast, a higher activity in the spatial than in the perceptual training group was not observed in any brain region. Importantly, there were no differences in brain activation during the maze task between the spatial and perceptual training groups at pretest. There were no differences in activation between the cycling and stretching groups, neither at pretest nor at posttest. The activity in the two physical training groups did not develop differently from pre- to posttest (lack of any interactions between the physical and the cognitive training).

**Table 3:** Spatial coordinates of the local maxima for the one-sample-t-Test showing activations during the experimental encoding phase compared to the control encoding phase at pretest (p < .05; FWE-/FDR-corrected; voxel per cluster > 9)

	**Coordinates (x, y, z in mm)**			**Voxel-level**	
**Region**	**Right hemisphere**	**Left hemisphere**	**Voxel per cluster**	***T***	***Z***
*Whole volume (FWE)-corrected*					
Inferior parietal cortex	42, -39, 42		175	9.08	6.34
Precuneus	6, -60. 53		427	11.64	7.23
Insula		−30. 21, 0	71	8.34	6.04
	30, 21, 4		263	10.90	6.99
SMA	6, 15, 46		93	8.25	6.00
Inferior frontal gyrus		−42, 18, 21	134	7.62	5.71
Middle frontal gyrus	36, 3, 53		148	8.00	5.89
		−30, 0, 53	43	7.97	5.87
	39, 39, 28		36	7.25	5.54
		−39, 36, 28	10	6.44	5.12
Middle temporal gyrus	45, -72, 21		60	7.65	5.73
Inferior temporal gyrus		−51, -54, -7	52	7.23	5.53
	51, -54, -7		33	6.86	5.34
Cerebellum		−30, -69, -25	27	7.52	5.67
Anterior cingulate cortex		−3, 6, 25	67	7.50	5.66
		−12, 27, 18	20	7.68	5.74
	15, 21, 25		58	7.14	5.48
*Small volume (FDR)-corrected*					
Medial frontal gyrus		−6, 18, 49	429	7.81	5.80
Inferior parietal cortex	39, -42, 42		241	8.49	6.10
		−36, -42, 39	511	6.97	5.40
Superior parietal cortex	18, -63, 49		244	8.54	6.12
		−21, -63, 56	296	6.42	5.11
Retrosplenial cortex	15, -54, 18		509	5.82	4.77
Cuneus	18, -60, 39		368	7.60	5.70
Caudate nucleus	15, 6, 14		132	7.09	5.46
		−15, -3, 18	129	7.84	5.82

**Table 4:** Spatial coordinates of the local maxima for the one sample t-Test showing activations during the experimental encoding phase compared to the control encoding phase at posttest (p < .05; FWE-/FDR-corrected; voxel per cluster > 9)

	**Coordinates (x, y, z in mm)**			**Voxel-level**	
**Region**	**Right hemisphere**	**Left hemisphere**	**Voxel per cluster**	***T***	***Z***
*Whole volume (FWE)-corrected*					
Inferior parietal cortex	42, -42, 42		34	6.55	5.18
Inferior frontal gyrus	48, 27, 25		30	6.74	5.28
	42, 12, 32		12	6.09	5.93
Middle frontal gyrus	39, 3, 53		67	7.39	5.61
	36, 39, 25		70	7.37	5.59
Superior frontal gyrus	21, 15, 53		24	6.86	5.34
*Small volume (FDR)-corrected*					
Medial frontal gyrus		−3, 15, 53	144	5.93	4.84
	27, 36. 32		39	4.90	4.20
	21, 0, 56		15	3.71	3.36
Inferior parietal cortex	39, -42, 42		210	6.32	5.06
		−36, -63, 49	114	3.98	3.56
Superior parietal cortex	39, -48, 56		167	5.48	4.57
		−12, -72, 53	94	4.87	4.18
Retrosplenial cortex	9, -54, 11		88	4.49	3.92
Caudate nucleus	15, 9, 14		110	4.25	3.76
		−15, 3, 14	89	4.13	3.67

**Table 5:** Spatial coordinates of the local maxima for the interaction Time × Cognitive training: Spatial/ T0 > Spatial/ T1 and Perceptual/ T0 < Perceptual/ T1 (p < .05; FDR-corrected; voxel per cluster > 9)

	**Coordinates (x, y, z in mm)**			**Voxel-level**	
**Region**	**Right hemisphere**	**Left hemisphere**	**Voxel per cluster**	***T***	***Z***
*Whole volume (FDR)-corrected*					
Middle temporal gyrus	48, -45, 7		18	4.84	4.42
Superior temporal gyrus	48, 12, -25		36	4.82	4.41
	36, 3, -18		33	4.55	4.20
Medial temporal lobe	39, -12, -11		16	4.80	4.39

**Table 6:** Spatial coordinates of the local maxima for the spatial training < perceptual training comparison at the posttest (p < .05; FDR-corrected; voxel per cluster > 9)

	**Coordinates (x, y, z in mm)**			**Voxel-level**	
**Region**	**Right hemisphere**	**Left hemisphere**	**Voxel per cluster**	***T***	***Z***
*Small volume (FDR)-corrected*					
Hippocampus	39, -12, -14		155	5.14	4.34
		−24, -9, -14	122	3.73	3.37
*Whole volume (FDR)-corrected*					
Superior frontal gyrus	15, 51, 39		63	3.93	3.51
	18, 30, 46		14	3.53	3.21
Inferior frontal gyrus	51, 36, -4		37	4.07	3.61
	45, 21, 18		17	3.77	3.39
Middle frontal gyrus		−27, 42, -7	19	3.90	3.49
		−39, 48, -11	14	3.63	3.28
		−33, 18, 49	12	3.50	3.19
Superior temporal gyrus	36, 3, -18		1441	6.19	4.96
	54, -51, 7		381	5.08	4.30
		−63, -24, -4	13	3.95	3.53
Middle temporal gyrus		−51, 9, -28	347	4.70	4.05
Parahippocampal gyrus	24, -51, -11		135	4.78	4.11
Precentral gyrus	45, -18, 56		34	3.79	3.41
Postcentral gyrus	30, -39, 49		16	3.66	3.31

## Discussion

The present controlled interventional study examined the effects of a cognitive intervention (spatial training vs. perceptual control training) and a physical intervention (endurance training vs. non-endurance training) on spatial learning and associated functional brain activations in healthy middle-aged adults. Spatial learning of a virtual maze tended to improve after a spatial training involving viewpoint shifting and path integration. Only the spatial training group showed significant changes in brain activation in the right middle and superior temporal gyrus and medial temporal lobe from pretest to posttest. At posttest, participants of the spatial training group showed lower activation levels than participants in the perceptual training group in a network of brain regions associated with spatial learning such as the hippocampus and parahippocampal gyrus. In contrast, the type of physical intervention did neither increase spatial training gains nor performance and associated brain activity in the virtual maze task. Thus, we did not find support for our hypothesis that cognitive and physical training would result in additive or even supra-additive gains for cognitive functioning.

Based on the findings in animals that both physical exercise and spatial training increased neuronal plasticity particularly in the hippocampus and performance in spatial memory tasks (e.g. [43]), performance in a spatial learning and navigation paradigm was chosen as the dependent variable in the present intervention study. This task was realized in a virtual maze task and required participants to build up a cognitive map of a virtual town. Only participants who took part in the spatial training, which involved viewpoint shifting and path integration tasks, were able to solve the virtual maze task at posttest and showed reduced brain activations at posttest compared to pretest. To successfully solve the virtual maze task, participants had to integrate the position of landmarks into a cognitive representation of the town and to continuously update their position in the environment by making use of visual motion cues. Thus, participants of the spatial learning group were able to transfer their newly acquired spatial abilities to the test task, i.e. the virtual maze task. This type of transfer has been called “near transfer” [75] and means that an ability is generalized from the training task, involving different stimuli and tasks, to a more complex test task of the same functional domain. In contrast, participants of the spatial training group did not outperform participants of the perceptual training group in tests of verbal learning and executive functions (‘far’ transfer). This result is in line with previous studies in humans (e.g. [36]) and animals [76], and suggests that spatial training induced changes limited to spatial processing systems. Furthermore, our results are in line with training studies in other cognitive domains, suggesting that a transfer from training to test is mainly observed when the training task and the test task depend on overlapping functions associated with similar brain regions [28]. It remains to be determined whether spatial skills acquired in a virtual environment would generalize to the real world as well, although there is evidence that virtually acquired spatial knowledge transfers at least to real-world settings that are similar to the learned virtual environment [77].

On the neuronal level, the spatial training group, as compared to the perceptual training group, showed significant activation changes from pre- to posttest in the superior and middle temporal gyri and the medial temporal lobe of the right hemisphere.

Lateral temporal cortex has been shown to be involved in declarative memory, both during encoding and retrieval [78]. Moreover, the middle and superior temporal gyri have been discussed as parts of a neural network which encodes spatial relations; especially the right temporal areas seem to be involved in calculating spatial coordinates [79]. Activations in these temporal lobe structures have been shown to correlate with participants’ performance during spatial navigation [40,58]. It might be speculated that brain activations in lateral temporal regions in the present study were involved in encoding the spatial relationship between buildings in the virtual maze tasks. A reduced activity in these areas at posttest for the spatial learning group might reflect a more efficient encoding of the spatial layout compared to posttest [38,39].

Furthermore, a comparison of the spatial and perceptual training group at posttest revealed a reduced activation in the spatial training group in a network of brain areas associated with spatial learning, including the hippocampus and parahippocampal gyrus.

Hippocampal and parahippocampal activity have been shown to correlate with navigation abilities [41], navigation strategies [80] and increasing practice [34]. Moreover, interindividual differences in navigational expertise have been linked to structural properties of the human hippocampus [81,82], at least in young adults [83]. Previous data using a similar spatial paradigm as in the present *f*MRI sessions had shown decreasing hippocampal activity with increasing knowledge of the environment [53].

Thus, as seen in the present study, the reduced activity in the spatial training group (as compared to the perceptual group) might reflect a better elaborated spatial knowledge.

Participants in the present sample (middle-aged men and women), however, did not reach a performance in the virtual maze task that was as high as the performance of the participants of Wolbers and Büchel [53]. We think this is mainly due to the age difference of the participants. Moreover, Wolbers and Büchel had recruited only male adults while both men and women were recruited for the present study. Although Wolbers and Büchel [53] provided evidence for reduced hippocampal activity along with superior spatial performance, it has to be noted that there are other studies which demonstrated increased hippocampal activity in participants with more successful spatial navigation skills (e.g. [84,85]). Moreover, age-related decline in spatial navigation performance has been associated with reduced hippocampal and parahippocampal activation [85,86]. Thus, future studies are necessary to disentangle more precisely how age, navigation performance and strategies affect functional brain activation patterns in the medial temporal lobe.

Reduced activations in the spatial training group, as compared to the perceptual group, were seen in the frontal cortex as well. The medial frontal gyrus has been associated with spatial short-term memory [66]. Moreover, the prefrontal cortex seems to play an important role for ‘higher cognitive processes’ in general and has been linked to the difficulty of a task [87]. A reduced activity in prefrontal areas subsequent to practice is a well known correlate of learning and has been interpreted as an increase in neural efficiency [39].

Although the spatial training group tended to improve in the virtual maze task, and showed significant changes in associated brain activations from pre- to posttest, the aerobic endurance training group did not gain more than the non-endurance control group. This held true for the learning improvements across training sessions as well. The result differs from reports of previous studies in older adults. They reported enhanced cognitive performance after an aerobic endurance training as compared to a non-endurance training (e.g. [17,19]). The sample in the present study was younger (40 to 55 years) than the samples of most previous studies (mostly older than 65 years). It has been discussed that beneficial effects of physical fitness on cognition increase with increasing age. In a meta-analysis, Colcombe and Kramer [18] provided evidence that participants between 66 and 70 years of age benefit more from exercise interventions than adults between 55 and 65 years of age. It might be speculated that beneficial effects of aerobic exercise on spatial learning are not yet observable in middle-aged adults, and may become evident only when these functions have already been subject of a larger degree of age-related decline. Although brain activations were not modulated by physical exercise on the group level, individual cardiovascular fitness and training induced fitness gains were positively correlated with brain activations during the virtual maze task for participants who took part in an additional spatial training (reported in [54]). The present results, together with our previous report, suggest that a spatial training has an immediate effect on neural networks associate with spatial learning (at least in middle-aged adults), whereas the influence of an endurance training might cause only subliminal effects on neurocognitive functioning, which are detected only in older age after age-related decline has further progressed. Indeed, prospective studies provided evidence that physical activity in midlife correlated with gray matter volume 20 years later [52] and participants that reported regular physical activity in midlife had a reduced risk of dementia at follow-up [10].

Another reason for the lack of a significant difference between the endurance and non-endurance training group might be that the stretching and coordination exercise in the present study promoted functions supporting spatial learning. Indeed Ruscheweyh et al. [20] demonstrated that physical activity has beneficial effects on memory functions independent of exercising intensity. Moreover, Voelcker-Rehage et al. [88] reported higher performance in older adults in a visual search task and in an executive task after a coordination training compared to a control group. *F*MRI data revealed changes in frontal and parietal areas, both after a cardiovascular training and a coordination training, suggesting that besides cardiovascular trainings, other types of exercise might have beneficial effects on cognition as well [89].

The sample size in the present study was rather small. On the behavioral level, effects of the spatial training showed only trends in the predicted direction. Moreover, null effects such as the non-significant interaction of physical and cognitive training must be interpreted with caution. The present results, together with our previous report of cardiovascular fitness modulating brain activations during a virtual spatial learning task [54], however, provide first empirical support in humans that the combination of a cognitive training with a physical intervention might promote neuronal plasticity in midlife. More studies with larger sample sizes should further address the question of additive or supraadditive effects of cognitive and physical trainings on cognitive functions in humans. Another limiting factor of the present study might be the rather overall low performance of participants in the maze task. This was unexpected given the results of previous studies with this paradigm [53,66] and, as discussed above, is most likely due to age differences between study samples. Spatial memory has been shown to decrease as early as in midlife [90]. Nevertheless, middle-aged adults still outperformed participants older than 55 years of age [91]. Future studies should use a test task better adapted for the age group under investigation.

## Conclusions

The present study demonstrated that a training of spatial abilities, compared to a perceptual training, modulated brain activations during spatial learning in a virtual maze task. After the cognitive intervention, brain activations were lower in the spatial training group than in the perceptual training group in a network of brain areas associated with spatial navigation, possibly demonstrating a more efficient neural processing. Moreover, the spatial training group tended to improve in the virtual maze task of spatial learning more than the perceptual training group. Hence, specific cognitive programs might be a useful tool to maintain and improve cognitive functions already in middle-aged adults.

## Endnotes

^a^As mentioned in the section "Participants", the *f*MRI data reported here was part of a larger intervention study investigating the effect of a physical training on cognition in humans. When analyzing data of all participants who took part in the spatial training (N = 34, N = 18 cycling group, N = 16 stretching group), the same pattern of results emerged: participants improved in viewpoint shifting and path integration across training sessions, but there was no difference between the cycling group and the stretching group (Training day × Physical intervention all p > 0.4). Thus, the lack of a significant interaction of group and time does not seem to be due to the small sample size of the *f*MRI sample.

^b^Learning score of the Auditory Verbal Learning Test: for the spatial training group: *M* = 60.0 (*SD* = 5.6) pretest vs. *M* = 65.9 (*SD* = 4.9) posttest; for the perceptual training group: *M* = 57.8 (*SD* = 8.7) pretest vs. *M* = 64.1 (*SD* = 7.1) posttest.

Reading time in sec for the Stroop task: for the spatial training group: *M* = 76.3 (*SD* = 13.2) pretest vs. *M* = 74.3 (*SD* = 17.6) posttest; for the perceptual training group: *M* = 74.8 (*SD* = 14.2) vs. *M* = 69.6 (*SD* = 11.7) posttest.

## Competing interests

The authors declare that they have no competing interests.

## Authors’ contribution

KH and KaHo contributed equally to the paper (shared first authorship). KH conceived the study, supervised the cognitive and physical trainings, analyzed the behavioral data and drafted the manuscript. KaHo carried out the fMRI-experiment, analyzed the fMRI data and drafted the manuscript. AS contributed to the design of the spatial training, carried out the cognitive training and analyzed parts of the behavioral data. TW provided the maze task and contributed to the analysis of the fMRI experiment. BR initiated and conceived the study and helped to draft the manuscript. All authors read and approved the final manuscript.
